# Developmental Changes in Expression of βIV Spectrin Splice Variants at Axon Initial Segments and Nodes of Ranvier

**DOI:** 10.3389/fncel.2016.00304

**Published:** 2017-01-10

**Authors:** Takeshi Yoshimura, Sharon R. Stevens, Cristophe Leterrier, Michael C. Stankewich, Matthew N. Rasband

**Affiliations:** ^1^Department of Neuroscience, Baylor College of MedicineHouston, TX, USA; ^2^Division of Neurobiology and Bioinformatics, National Institute for Physiological Sciences, National Institutes of Natural SciencesOkazaki, Japan; ^3^CNRS, Center for Research in Neurobiology and Neurophysiology of Marseille (CRN2M) UMR 7286, Aix Marseille UniversitéMarseille, France; ^4^Department of Pathology, Yale UniversityNew Haven, CT, USA

**Keywords:** cytoskeleton, spectrin, axon, node of Ranvier, axon initial segment, ankyrin

## Abstract

Axon initial segments (AIS) and nodes of Ranvier are highly specialized axonal membrane domains enriched in Na^+^ channels. These Na^+^ channel clusters play essential roles in action potential initiation and propagation. AIS and nodal Na^+^ channel complexes are linked to the actin cytoskeleton through βIV spectrin. However, neuronal βIV spectrin exists as two main splice variants: a longer βIVΣ1 variant with canonical N-terminal actin and αII spectrin-binding domains, and a shorter βIVΣ6 variant lacking these domains. Here, we show that the predominant neuronal βIV spectrin splice variant detected in the developing brain switches from βIVΣ1 to βIVΣ6, and that this switch is correlated with expression changes in ankyrinG (ankG) splice variants. We show that βIVΣ1 is the predominant splice variant at nascent and developing AIS and nodes of Ranvier, but with increasing age and in adults βIVΣ6 becomes the main splice variant. Remarkably, super-resolution microscopy revealed that the spacing of spectrin tetramers between actin rings remains unchanged, but that shorter spectrin tetramers may also be present. Thus, during development βIV spectrin may undergo a switch in the splice variants found at AIS and nodes of Ranvier.

## Introduction

Neurons are highly polarized cells comprised of two structurally and functionally distinct domains: the axon and dendrites (Craig and Banker, 1994). The polarization of neurons allows for the unidirectional flow of information from dendrites/soma to axons. The dendrites and soma receive the upstream synaptic inputs; these are integrated and the decision to fire an action potential is made at the axon initial segment (AIS; Yoshimura and Rasband, 2014). AIS are characterized by high densities of voltage-gated Na^+^ and K^+^ channels that function to initiate and modulate action potentials (Kole and Stuart, 2012). In myelinated axons of vertebrates, action potentials propagate through the opening of Na^+^ channels at nodes of Ranvier.

The AIS and nodes of Ranvier have a common molecular organization; in addition to the clustering of Na^+^ and K^+^ channels, these two regions share an enrichment of adhesion molecules and molecular scaffolds (Chang and Rasband, 2013). AnkyrinG (ankG) and βIV spectrin are the main components of the cytoskeleton at the AIS and nodes of Ranvier in neurons (Kordeli et al., 1995; Berghs et al., 2000). AnkG interacts with and clusters membrane proteins (e.g., Na+ channels) and βIV spectrin, while βIV spectrin is thought to link the ankG/Na+ channel membrane protein complex to the actin cytoskeleton (Yang et al., 2007; Ho et al., 2014). Loss of ankG disrupts AIS assembly and neuronal function (Zhou et al., 1998). In βIV spectrin deficient mice, ankG and Na^+^ channel densities are reduced at the AIS, probably due to diminished stability of the complex (Komada and Soriano, 2002). Thus, both ankG and βIV spectrin are essential for proper AIS ion channel complexes and axon domain organization.

**Figure 1:**
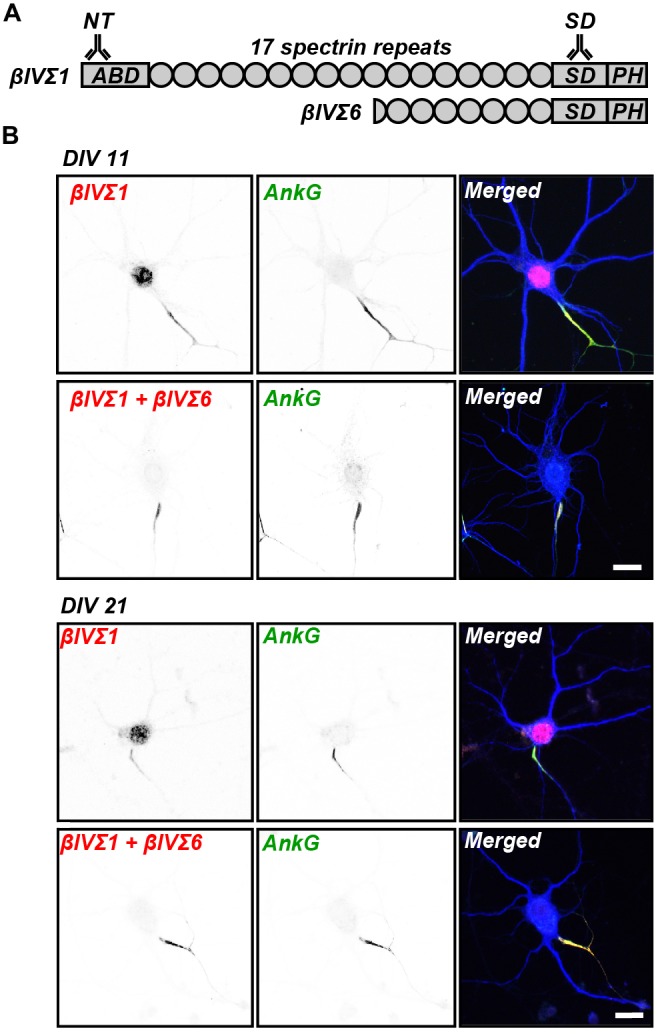
**βIV spectrin splice variants. (A)** Two βIV spectrin splice variants, Σ1 and Σ6, are found at the axon initial segments (AIS) and nodes of Ranvier. These proteins have multiple spectrin repeats (SRs), βIVΣ1 has an actin binding domain (ABD), and both have specific domains (SD) and pleckstrin homology (PH) domains. Antibodies against the NT and SD domains can be used to detect these splice variants. **(B)** Immunostaining of cultured hippocampal neurons at days *in vitro* (DIV) 11 and 21 using NT (βIVΣ1) and SD (βIVΣ1 + βIVΣ6) antibodies labels AIS. AIS are defined by the immunostaining for AnkyrinG (ankG). MAP2, a somatodendritic marker, is shown in blue in the merged images. Scale bar = 20 μm.

Alternative splicing generates six βIV spectrin splice variants (βIVΣ1–βIVΣ6; Berghs et al., 2000; Komada and Soriano, 2002). Two variants, βIVΣ1 and βIVΣ6, are thought to be at the AIS and nodes of Ranvier (Komada and Soriano, 2002; Lacas-Gervais et al., 2004). βIVΣ1 is the largest of the splice variants and consists of an N-terminal actin-binding domain, 17 spectrin repeats (SRs), a “specific” domain (SD) and a pleckstrin homology (PH) domain (Figure 1A). βIVΣ6 is an N-terminal truncation that lacks the actin-binding domain, SRs 1–9, and part of SR 10 (Figure 1A). Spectrins are thought to function as heterotetramers consisting of two α and two β spectrin subunits, with the minimal lateral interactions occurring between the first two triple-helical domains of β-spectrins and the last two triple helical domains of α-spectrins (Speicher et al., 1992). Thus, it is not clear how βIVΣ6 would function in a heterotetramer. Due to the poorly understood expression patterns of the βIV spectrin splice variants, their individual roles remain unclear. Although the ~190 nm spacing of βIV spectrin as revealed by super-resolution microscopy suggests they function as tetramers in the AIS, no α spectrin has yet been reported at the AIS (Galiano et al., 2012; Xu et al., 2013; Leterrier et al., 2015).

Here, we examined the spatial and temporal expression patterns of βIVΣ1 and βIVΣ6 at the AIS and nodes of Ranvier. We used anti-βIV spectrin antibodies against the N-terminal region that detects βIVΣ1, and antibodies against the SD that detects both βIVΣ1 and βIVΣ6 (Figure 1A). We found in early development that βIVΣ1 is the dominant splice variant at the AIS and nodes of Ranvier. However, as development proceeded, this shifted and βIVΣ6 became the dominant isoform in adult mice. Finally, stochastic optical reconstruction microscopy (STORM) super-resolution microscopy revealed that the pattern of βIV spectrin immunoreactivity remained periodic despite the switch in expression of these two splice variants, although a more complicated periodic pattern suggests the existence of shorter spectrin tetramers that may include βIVΣ6. Together, these studies reveal the differential expression and localization of βIV spectrin splice variants at the AIS and nodes of Ranvier during development of the nervous system.

## Materials and Methods

### Animals

Sprague Dawley rats were obtained from Harlan Sprague Dawley (Indianapolis, IN, USA). C57BL/6 mice were from Charles River Laboratories (Wilmington, MA, USA). Both male and female mice were used in these studies. All experiments were performed in compliance with the National Institutes of Health Guide for the Care and Use of Laboratory Animals and were approved by the Baylor College of Medicine Institutional Animal Care and Use Committee.

### Antibodies

Rabbit anti-βIV spectrin NT, and rabbit or chicken anti-βIV spectrin SD antibodies were previously described (Yang et al., 2004). The anti-βIV spectrin NT antibody was generated against a synthetic peptide corresponding to amino acids 15–38 of the βIVΣ1 splice variant. Anti-βIV spectrin SD antibody was generated against the SD (Berghs et al., 2000). The goat anti-ankG antibody for immunoblot analysis was generated against the C-terminal domain of ankG and was kindly provided by Dr. Vann Bennett (Duke University, Durham, NC, USA; Ho et al., 2014). The following other primary antibodies were used: mouse monoclonal anti-ankG for immunostaining (N106/36, UC Davis/NIH NeuroMab facility, Davis, CA, USA); rabbit anti-GAPDH (Sigma-Aldrich, St. Louis, MO, USA); mouse monoclonal anti-Caspr (K65/35, UC Davis/NIH NeuroMab facility); and chicken polyclonal anti-MAP2 (EnCor Biotechnology, Gainesville, FL, USA) antibodies. Secondary antibodies were purchased from Jackson ImmunoResearch Laboratories (West Grove, PA, USA) and Life Technologies (Thermo Fisher Scientific, Waltham, MA, USA).

### Brain Lysate Preparation

Whole mouse brains were dissected and homogenized in ice-cold homogenization buffer (0.32M sucrose, 5 mM sodium phosphate, pH 7.4, 1 mM sodium fluoride and 1 mM sodium orthovanadate, containing 0.5 mM phenylmethylsulfonyl fluoride, 2 μg/ml aprotinin, 1 μg/ml leupeptin, 2 μg/ml antipain and 10 μg/ml benzamidine). Crude homogenates were then centrifuged at 600× g for 10 min to remove debris and nuclei. The resulting supernatant was centrifuged at 30,000× g for 90 min, then the pellet was re-suspended in ice-cold homogenization buffer. Protein concentrations were determined using the BCA method (Thermo Fisher Scientific, Waltham, MA, USA). After adding SDS sample buffer, the samples were subjected to SDS–PAGE and immunoblot analysis.

### RT-qPCR

RNA was isolated from whole mouse brains in triplicate using Trizol (Invitrogen, Carlsbad, CA, USA) and converted to cDNA with Superscript (Invitrogen) using a combination of oligo(T)s and random hexamers. mRNA was quantified with a Nanodrop spectrophotometer to ensure equal starting amounts. Gene specific primers that bridged consecutive exons were used to ensure detection of mRNA. For qPCR, using Power SYBR^®^ Green PCR Master Mix (Applied Biosystems), the signal from every reaction at the end of each 60°C annealing extension step of each cycle was recorded on a CFX96 Real Time System (BioRad). Data are normalized to GAPDH. Primers used are as follows: βIVΣ1 (forward) TGAGGGCCCAGCAGTGGATGC; βIVΣ6 (forward) GACGCTCCTCCAACGCCG; βIVΣ1/Σ6 (reverse) GGTGCCGGAGCCATCTCTTGT; 190 AnkG (forward) CTTTGCCTCCCTAGCTTTAC; 190 AnkG (reverse) TCTGTCCAACTAAGTCCCAG; 270 AnkG (forward) GCCATGTCTCCAGATGTTG; (reverse) TCTGTCCAACTAAGTCCCAG; 480 AnkG (forward) AGTAGGAGGACTGGTCCG; (reverse) AGTTGTGGCATTCTTTCCG.

### Culture of Hippocampal Neurons

Brains from embryonic day 18 rat embryos were collected into ice-cold HBSS without calcium or magnesium (Invitrogen, Carlsbad, CA, USA). Embryonic hippocampi were dissected and collected in ice-cold HBSS. The collected tissue was incubated with 0.25% Trypsin in HBSS at 37°C for 15 min and washed with HBSS. After adding plating media (Neurobasal medium (Invitrogen) with 10% HyClone FetalClone III serum (Thermo Fisher Scientific, Waltham, MA, USA)), these hippocampi were mechanically dissociated using a fire-polished Pasteur pipette. The suspension was centrifuged for 5 min at 200× g. The pelleted cells were briefly washed and resuspended in plating media. Neurons were plated on glass coverslips coated with poly-D-lysine (Sigma-Aldrich) at low density for immunocytochemistry (120 cells/mm^2^) and on plastic dishes with poly-D-lysine at high density for western blot (520 cells/mm^2^). After neurons were incubated in a humidified 5% CO_2_ incubator at 37°C for 3 h, the media was exchanged to maintaining media (Neurobasal medium with 2% B-27 supplement (Invitrogen) and 2 mM GlutaMAX (Invitrogen)). The cultures were maintained by exchanging half of the volume of media twice a week with new maintaining media. For immunoblotting, neurons were washed with PBS, and collected with SDS sample buffer. The samples were subjected to SDS–PAGE and immunoblot analysis.

### Immunostaining and Imaging

Cultured neurons at 11 and 21 days *in vitro* (DIV) were fixed in 4% paraformaldehyde (PFA) in 0.1 M phosphate buffer (PB, pH 7.2) at 4°C for 30 min, followed by treatment with PBTGS (0.1M PB with 0.3% Triton X-100 (Sigma-Aldrich) and 10% goat serum (Invitrogen)) for 1 h at room temperature. For immunostaining of nervous system tissues, brains, optic and sciatic nerves were dissected at the indicated times, fixed in 4% PFA for 30 min for optic and sciatic nerves, or 1.5 h for brains; this was followed by immersion in 20% sucrose (w/v) in 0.1 M PB overnight at 4°C. The tissues were then frozen in Tissue-Tek OCT compound (Sakura Finetek, Tokyo, Japan) and sectioned using a Cryostat (CryoStar NX70, Thermo Fisher Scientific). Sections were collected and suspended in 0.1 M PB, then spread out on glass coverslips. The tissues were treated with PBTGS for 1 h at room temperature. The samples were incubated with primary antibodies diluted in PBTGS at room temperature overnight. Following this, samples were incubated with secondary antibodies for 1 h at room temperature. Immunofluorescence labeling was visualized, and images were collected on an AxioImager Z1 microscope (Carl Zeiss, Jena, Germany) fitted with an AxioCam digital camera (Carl Zeiss). AxioVision (Carl Zeiss) software was used for the collection and measurement of images. Images used for fluorescent signal quantification were collected using the same exposure settings across all animals and stages for the channels with βIV spectrin NT and βIV spectrin SD.

### STORM Imaging

After 13–28 days in culture, neurons were fixed using 4% PFA for 10 min. After blocking, they were incubated with primary antibodies overnight at 4°C, then with secondary antibodies for 1 h at room temperature. STORM imaging was performed on an N-STORM microscope (Nikon Instruments, Melville, NY, USA). Coverslips were imaged in STORM buffer: Tris 50 mM (pH 8); NaCl 10 mM; 10% glucose; 100 mM MEA; 3.5 U/ml pyranose oxidase; and 40 mg/ml catalase. The sample was continuously illuminated at 647 nm (full power) and 30,000–60,000 images were acquired at 67 Hz, with progressive reactivation by simultaneous 405-nm illumination (Leterrier et al., 2015).

### Image Quantification

Images were analyzed using NIH ImageJ software. Mean fluorescent signal intensities of rabbit anti-βIV spectrin NT and chicken anti-βIV spectrin SD were quantified in raw images using a line scan along the length of the AIS or for a region of interest encompassing the area of the node of Ranvier. All visible AIS or nodes of Ranvier were measured in each image; these measurements were averaged for individual animals. Population means were calculated for all tissues and developmental stages. Measurements for the rabbit anti-βIV spectrin NT antibody showed expression of βIVΣ1; the difference of the fluorescence intensities for the two βIV spectrin antibodies gave the relative expression of βIVΣ6. Three animals were used at each time point analyzed. All statistical comparisons were performed using Student’s *t*-test. The number of AIS measured: P1 (*n* = 96), P3 (*n* = 96), P9 (*n* = 96), 5-mo (*n* = 56). Number of nodes of Ranvier measured in optic nerves: P9 (*n* = 115), P15 (*n* = 80), P30 (*n* = 80), 5-mo (*n* = 80). Number of nodes of Ranvier measured in sciatic nerves: P1 (*n* = 120), P3 (*n* = 120), P9 (*n* = 118), 5-mo (*n* = 80). Number of nodes of Ranvier measured in cerebellum: P9 (*n* = 141), P15 (*n* = 90), P30 (*n* = 102), 5-mo (*n* = 114).

## Results

### βIVΣ6 Is Highly Upregulated Following AIS Formation

Cultured hippocampal neurons are commonly used to study the development of neuronal polarity and assembly of unique domains (e.g., synapses, AIS, etc.; Galiano et al., 2012). To investigate the spatial expression patterns of βIV spectrin splice variants in neurons, we used two anti-βIV spectrin antibodies generated against the NT and SD domains (Figure 1A; Yang et al., 2004). The anti-βIV spectrin SD antibody recognizes both Σ1 and Σ6 variants, whereas the anti-βIV spectrin NT antibody recognizes only the Σ1 variant (Figure 1A). Immunostaining 11 and 21 DIV cultured hippocampal neurons with these antibodies showed both the SD and NT immunoreactivities colocalized with ankG at the AIS (Figure 1B). Since only the NT antibody showed any staining in the nucleus, but not the SD antibody that recognizes both splice variants, we conclude the nuclear NT immunoreactivity is nonspecific.

**Figure 2:**
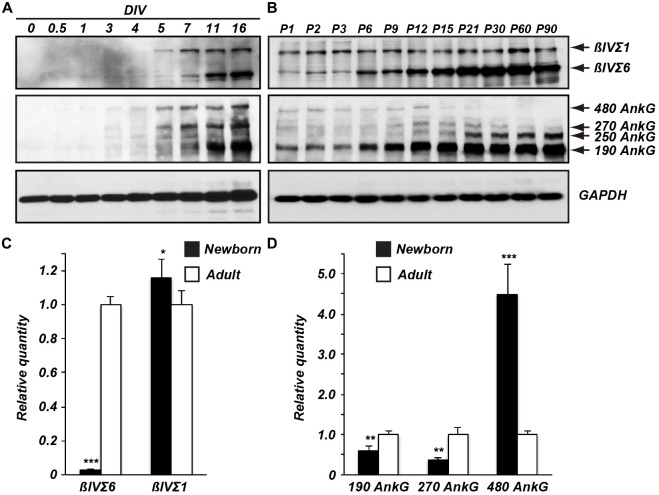
**Temporal expression of βIV spectrin and ankG splice variants.** Immunoblots of βIV spectrin and ankG from cultured neurons **(A)** and from brain homogenates **(B).** Reverse transcription-quantitative polymerase chain reaction (RT-qPCR) of βIV spectrin **(C)** and ankG **(D)** splice variant transcripts detected in developing brain. Error bars ± SEM. *p < 0.01, **p < 0.001, ***p < 0.0001.

To determine the temporal expression profile of βIV spectrin splice variants, we performed immunoblotting using homogenates of cultured hippocampal neurons and developing brain (Figures 2A,B). Both in cultured neurons and in developing mouse brain the βIVΣ1 splice variant was the earliest splice variant observed. However, the expression levels of βIVΣ6 dramatically increased at later time points both in culture and in the brain, indicating the most abundant form of βIV spectrin in mature neurons is the shorter βIVΣ6 splice variant.

βIV spectrin is localized to the AIS through the binding of its 15th SR to ankG; unlike the actin-binding-domain, the ankG-binding domain is present in both βIVΣ1 and βIVΣ6 (Figure 1A; Yang et al., 2007). We examined the expression levels of ankG in cultured hippocampal neurons and in developing mouse brain (Figures 2A,B). The main ankG splice variants thought to be located at AIS and nodes of Ranvier are the 480 and 270 kDa isoforms (Kordeli et al., 1995). In cultured neurons all ankG splice variants increased with age (Figure 1A). In contrast, in the brain there was a dramatic increase in the ankG 190 kDa isoform, and an apparent reduction in 480 and 270 kDa splice variants. Although these same antibodies robustly label AIS in brain sections (data not shown), we speculate the apparent reduction in 480 and 270 kDa forms may reflect their extreme detergent insolubility and poor resolvability by SDS-PAGE.

To further define the differential expression of the βIV spectrin and ankG splice variants, we measured their transcript levels by reverse transcription-quantitative polymerase chain reaction (RT-qPCR; Figures 2C,D). Consistent with the immunoblotting results, the βIVΣ6 transcript levels from adult mice were significantly higher than those from newborn mice (Figure 2C). In contrast βIVΣ1 transcript levels from newborn mice were slightly higher when compared to adult mice. AnkG transcripts from adult mice showed that levels of ankG 190 and 270 kDa were increased relative to newborn mice, while the levels of ankG 480 transcripts were significantly lower than in newborn mice. These results indicate that transcript and protein expression levels of βIV spectrin and ankG splice variants reflect similar changes during mouse brain development. In particular, βIVΣ6 is dramatically increased during development, while 480 kDa ankG is reduced.

### βIV Spectrin Splice Variants Change at the AIS and Nodes of Ranvier during Development

**Figure 3:**
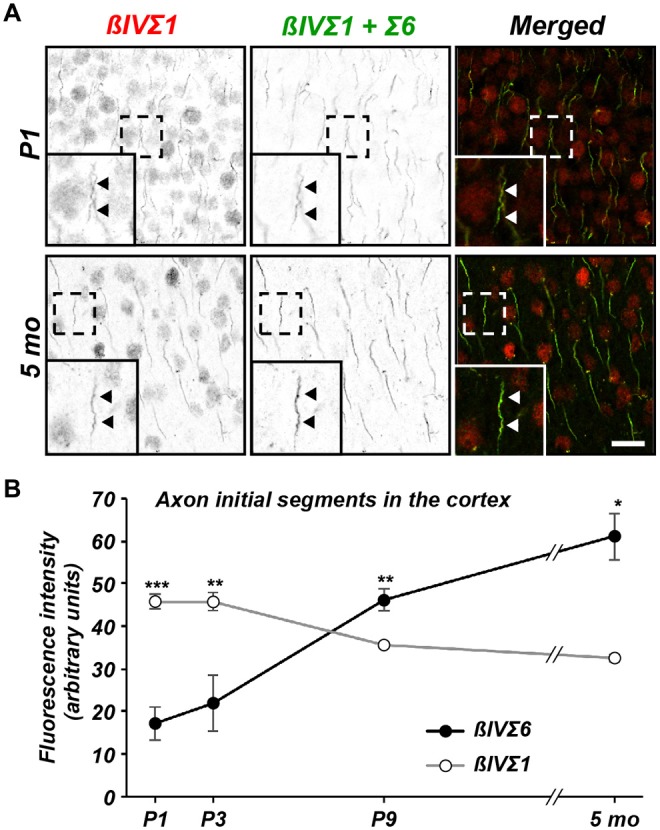
**Differential expression of βIV spectrin splice variants at cortical AIS. (A)** Immunolabeling of cortical AIS using NT (βIVΣ1) and SD (βIVΣ1 + βIVΣ6) antibodies in P1 and 5 month-old brain. Scale bar = 10 μm. **(B)** Quantification of relative βIVΣ1 and βIVΣ6 expression at the AIS in sections of cortex as a function of age. Error bars ± SEM. *p < 0.01, **p < 0.001, ***p < 0.0001.

To determine if the change in βIV spectrin splice variant expression occurs specifically at AIS, we compared the expression levels of βIVΣ1 and βIVΣ6 at AIS in mouse cortex throughout development by double-immunolabeling with rabbit anti-NT (βIVΣ1) and chicken anti-SD (βIVΣ1 + Σ6) antibodies. The SD immunoreactivity was localized at the AIS in both P1 and 5 month-old mouse cortex (Figure 3A). The NT immunoreactivity also labeled AIS and colocalized with the SD staining (Figure 3A; except for the non-specific nuclear immunoreactivity). Keeping all imaging parameters constant for each time point measured, we then subtracted the measured NT fluorescence intensity (βIVΣ1) from the measured SD fluorescence intensity (βIVΣ1 + Σ6) which permitted us to estimate the relative contribution of Σ6 to the SD immunostaining. We found that during early AIS assembly, βIVΣ1 is the predominant splice variant at AIS, but that this gradually changes such that by 5 months of age βIVΣ6 increases at the AIS relative to βIVΣ1 (Figure 3B).

**Figure 4:**
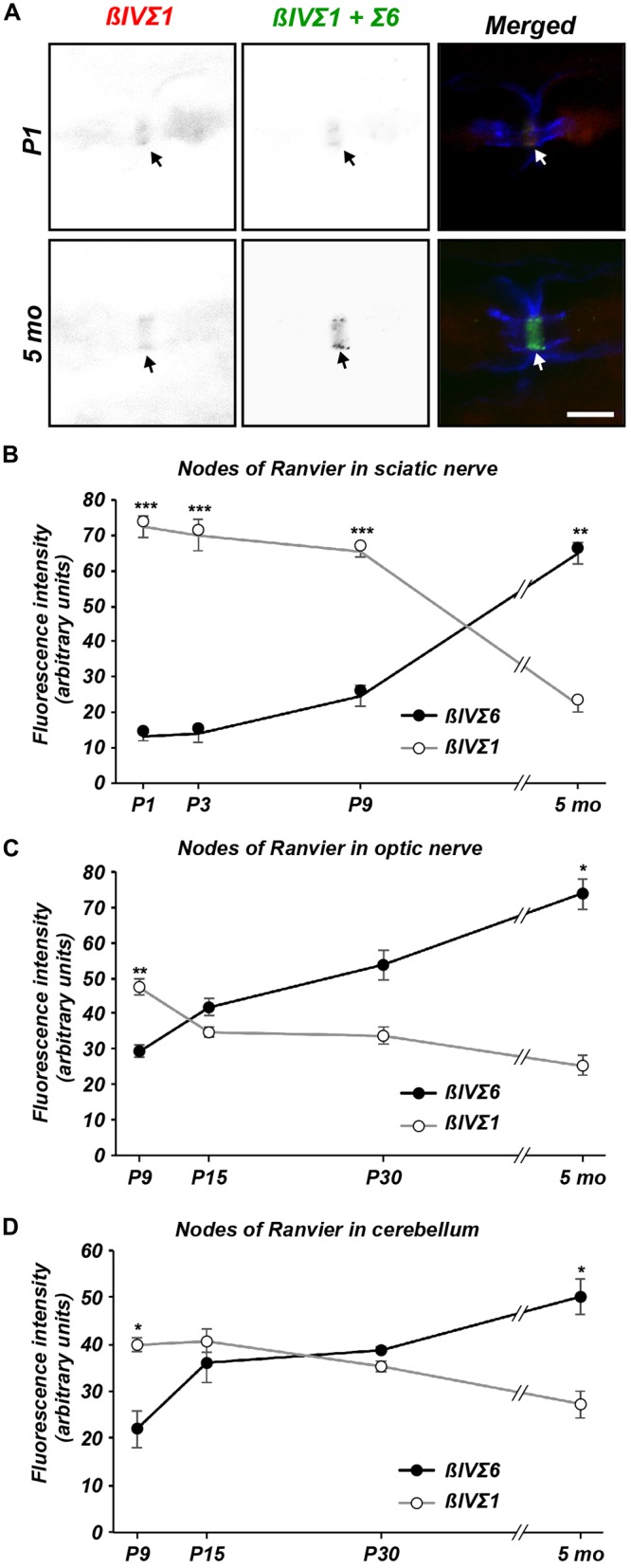
**Differential expression of βIV spectrin splice variants at nodes of Ranvier. (A)** Immunolabeling of nodes of Ranvier using NT (βIVΣ1) and SD (βIVΣ1 + βIVΣ6) antibodies in P1 and 5 sciatic nerve. Caspr immunostaining is shown in blue. Scale bar = 5 μm. **(B–D)** Quantification of relative βIVΣ1 and βIVΣ6 expression in sciatic nerve **(B)**, optic nerve **(C)** and cerebellar nodes **(D)** as a function of age. Error bars ± SEM. *p < 0.01, **p < 0.001, ***p < 0.0001.

Next, we performed a similar comparison of βIVΣ1 and βIVΣ6 immunostaining at nodes of Ranvier in sciatic nerve (Figures 4A,B), optic nerve (Figure 4C), and in cerebellum (Figure 4D). Remarkably, we found a similar shift at nodes from βIVΣ1 at new nodes of Ranvier to βIVΣ6 in older animals. These results suggest that while βIVΣ1 is the main βIV spectrin splice variant found at the AIS and nodes of Ranvier during early development, with increasing age βIVΣ1 may be replaced by the shorter βIVΣ6 splice variant. These observations are consistent with the results obtained by immunoblotting and RT-qPCR (Figure 2).

### Super-Resolution Imaging of βIV Spectrin Distribution during Development

**Figure 5:**
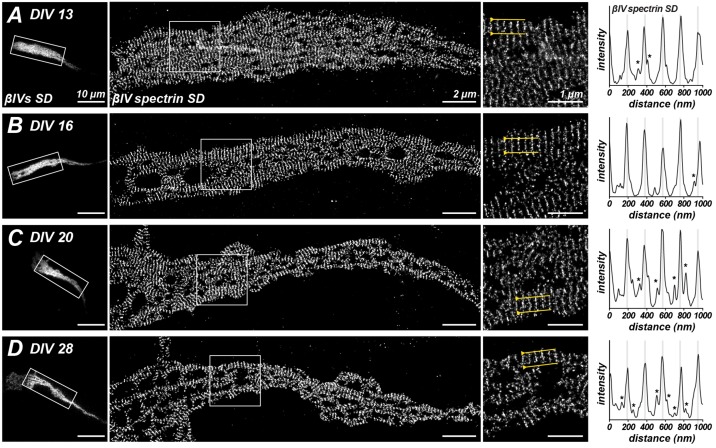
**Stochastic optical reconstruction microscopy (STORM) imaging of βIV SD localization at different ages in culture. (A–D)** Immunostaining for βIV spectrin SD at the AIS of cultured hippocampal neurons at DIV 13, 16, 20 and 28. Boxed regions correspond to magnified images at the right. Intensity profiles at the far right of each panel and correspond to regions between the yellow lines in the magnified images at the right. Lower intensity peaks, possibly corresponding to βIVΣ6 splice variants, in line scans are indicated by an asterisk. Scale bars = 10 μm (left widefield images), 2 μm (STORM images), 1 μm (right magnified images).

Recent experiments using STORM super-resolution microscopy revealed a periodic cytoskeleton at the AIS consisting of rings of actin spaced at ~190 nm intervals (Xu et al., 2013; Leterrier et al., 2015). Intriguingly, the spacing of these rings corresponds to the length of spectrin tetramers. Indeed, STORM imaging of βIV spectrin using our NT and SD antibodies revealed that the actin rings colocalized or alternate with the spectrin immunoreactivity, respectively (Xu et al., 2013; Leterrier et al., 2015). Although the spacing of AIS actin rings has not yet been shown to depend on βIV spectrin, the spacing of actin rings in the distal axon does depend on βII spectrin (Zhong et al., 2014). Since the lengths of βIVΣ1 and βIVΣ6 are quite different, we wondered if the spacing of actin rings might change during development with the increased abundance of the shorter splice variant at AIS. To test this possibility, we performed STORM imaging using SD antibodies on cultured hippocampal neurons at DIV 13, 16, 20 and 28 (Figures 5A–D). Despite the increased protein and expression of the shorter βIVΣ6 splice variant, we still observed that the major peak of SD immunoreactivity occurred at an interval of ~190 nm in older neurons, suggesting that the spacing continues to be determined primarily by the βIVΣ1 splice variant. Nevertheless, secondary peaks of lesser intensity could be detected on intensity profiles that were 55 ± 2.67 nm (SEM) to each side of the major peaks (see Figure 5C, asterisks). Importantly, these secondary peaks became more frequent and pronounced with increasing age.

## Discussion

βIV spectrin is highly enriched at the AIS and nodes of Ranvier and functions to link membrane proteins to the actin-based cytoskeleton through ankG (Berghs et al., 2000; Komada and Soriano, 2002; Yang et al., 2007). Mouse mutants of βIV spectrin have a myriad of nervous system abnormalities including disrupted nodes and AIS, hearing loss, ataxia, and even axon degeneration (Parkinson et al., 2001; Yang et al., 2004). Here, we showed that a shift from βIVΣ1 to βIVΣ6 occurs at the AIS and nodes of Ranvier during development, with βIVΣ1 being dominant in early development, but βIVΣ6 being dominant in mature neurons.

Spectrin α and β subunits form antiparallel dimers that self-associate to form tetramers of ~190 nm in length, and that function as a submembranous scaffolding network anchored to actin filaments by β spectrins (Bennett and Healy, 2008). In axons, the spectrin-actin cytoskeleton forms a periodic network of circumferential actin rings along the inner cytoplasmic face of the axon membrane; these actin rings are evenly spaced along axonal shafts by spectrin tetramers. This network has been proposed to participate in both the polarization of the AIS membrane and to provide structural support to axons to withstand mechanical stresses (Xu et al., 2013). Despite the canonical view of spectrin tetramers, the composition of the AIS spectrin cytoskeleton remains an enigma since: (1) the α spectrin subunit that partners with βIV spectrin has not yet been reported; and (2) βIVΣ6 lacks both the actin and α spectrin-binding domains. Despite our observation that βIVΣ6 is the main splice variant in mature neurons, the length of spectrin tetramers, as measured using SD antibodies, remained constant at about 190 nm. In addition, intensity profiles along the AIS revealed secondary peaks in SD immunoreactivity that may correspond to spectrin tetramers that include a βIVΣ6 subunit. Thus, it is possible that a single AIS (or node) spectrin tetramer consists of both βIVΣ1 and βIVΣ6 subunits. While co-immunoprecipitation experiments using expression constructs in heterologous cells may be able to determine if these splice variants can function in the same tetramer, the extreme detergent insolubility of the AIS makes it difficult to confirm that these tetramers of mixed splice variants exist in the brain. It is possible that βIV spectrin splice variants can associate with α spectrins through previously unidentified interacting domains present in both βIVΣ1 and βIVΣ6. Future studies of mice lacking βIVΣ1 using STORM might provide key insights into how the periodic spectrin cytoskeleton forms and is maintained, and how βIVΣ6 interacts with the actin cytoskeleton. Furthermore, it will be important to determine if α spectrin functions at the AIS in concert with βIV spectrin splice variants.

Different Na^+^ channels occupy distinct subdomains of the AIS. In cortical pyramidal neurons, Nav1.2 and Nav1.6 are found in the proximal and distal regions of the AIS, respectively (Hu et al., 2009). Furthermore, during development Nav1.2 is first found at nodes of Ranvier, but then as neurons mature, Nav1.6 replaces these channels. The switch in the type of nodal Na+ channels correlates with the onset of myelination and the sustained expression of Nav1.6 depends on myelination. Although the distributions of βIVΣ1 and βIVΣ6 are similar in the proximal and distal regions of the AIS, the switch in βIV spectrin splice variants was also coincident with the onset of myelination. However, since the switch in splicing also occurs in cultured hippocampal neurons that lack myelin, the change in splice variant expression does not depend on myelination. Furthermore, the switch from Nav1.2 to Nav1.6 is unlikely to depend on the change in βIV spectrin splice variant expression since Na^+^ channel binding to ankG is both necessary and sufficient for their AIS and nodal localization (Garrido et al., 2003; Gasser et al., 2012).

Why is there an increase in the amount of βIVΣ6 expression at AIS and nodes with increasing age? Although our experiments cannot answer this, it is unlikely that replacing βIVΣ1 with βIVΣ6 reflects the recruitment of additional proteins to the AIS since all of the protein coding regions found in βIVΣ6 are also found in βIVΣ1. Instead, we speculate that a decrease in βIVΣ1 may reflect a maturation of the AIS and loss of some proteins that associated with the N-terminal half of βIVΣ1 during AIS assembly or establishment of the trafficking filter associated with the AIS.

In conclusion, using two kinds of anti-βIV spectrin antibodies, NT for βIVΣ1 and SD for both βIVΣ1 and βIVΣ6 splice variants, we found a switch in the expression levels and localization of these splice variants at the AIS and nodes of Ranvier during development. Additional experiments will be required to determine the role this switch has for nervous system function.

## Author Contributions

TY and SRS performed immunoblots, immunostaining and quantification of signals. CL performed STORM imaging. MCS performed RT-qPCR. MNR and MCS conceived and directed the project. All authors contributed to writing and revising of the final manuscript.

## Funding

This work was supported by National Institutes of Health (NIH) grants NS044916 and the Dr. Miriam and Sheldon G. Adelson Medical Research Foundation. CL acknowledges B. Dargent for support, through grant ANR-2011-BSV4-001-1 by Agence Nationale de la Recherche.

## Conflict of Interest Statement

The authors declare that the research was conducted in the absence of any commercial or financial relationships that could be construed as a potential conflict of interest.
